# The FlhA linker mediates flagellar protein export switching during flagellar assembly

**DOI:** 10.1038/s42003-021-02177-z

**Published:** 2021-05-31

**Authors:** Yumi Inoue, Miki Kinoshita, Mamoru Kida, Norihiro Takekawa, Keiichi Namba, Katsumi Imada, Tohru Minamino

**Affiliations:** 1 Graduate School of Frontier Biosciences, Osaka University, Suita, Osaka Japan; 2 Department of Macromolecular Science, Graduate School of Science, Osaka University, Toyonaka, Osaka Japan; 3 RIKEN SPring-8 Center and Center for Biosystems Dynamics Research, Suita, Osaka Japan; 4 JEOL YOKOGUSHI Research Alliance Laboratories, Osaka University, Suita, Osaka Japan; 5 Present Address: Department of Ophthalmology and Visual Sciences, Kyoto University Graduate School of Medicine, Kyoto, Japan

**Keywords:** Bacterial secretion, X-ray crystallography, Motor protein structure

## Abstract

The flagellar protein export apparatus switches substrate specificity from hook-type to filament-type upon hook assembly completion, thereby initiating filament assembly at the hook tip. The C-terminal cytoplasmic domain of FlhA (FlhA_C_) serves as a docking platform for flagellar chaperones in complex with their cognate filament-type substrates. Interactions of the flexible linker of FlhA (FlhA_L_) with its nearest FlhA_C_ subunit in the FlhA_C_ ring is required for the substrate specificity switching. To address how FlhA_L_ brings the order to flagellar assembly, we analyzed the *flhA(E351A/W354A/D356A)* Δ*flgM* mutant and found that this triple mutation in FlhA_L_ increased the secretion level of hook protein by 5-fold, thereby increasing hook length. The crystal structure of FlhA_C_(E351A/D356A) showed that FlhA_L_ bound to the chaperone-binding site of its neighboring subunit. We propose that the interaction of FlhA_L_ with the chaperon-binding site of FlhA_C_ suppresses filament-type protein export and facilitates hook-type protein export during hook assembly.

## Abstract (web summary)

Inoue and Kinoshita et al. report an important role for the flexible linker of the major flagellar export apparatus protein FlhA (FlhA_L_) in regulating the switch from secretion of hook-type to filament-type components. The data presented here improve our understanding of the complex process of bacterial flagellar assembly.

## Introduction

The flagellum of *Salmonella enterica* (hereafter referred to as *Salmonella*) is a supramolecular motility machine consisting of the basal body, which acts as a bi-directional rotary motor, the hook, which functions as a universal joint, and the filament, which works as a helical propeller^1^. For construction of the flagella on the cell surface, the flagellar type III secretion system (fT3SS) transports flagellar building blocks from the cytoplasm to the distal end of the growing flagellar structure^2^. The fT3SS is divided into three structural parts: a transmembrane export gate complex made of FlhA, FlhB, FliP, FliQ, and FliR, a substrate-chaperone-docking platform composed of the cytoplasmic domains of FlhA and FlhB (FlhA_C_ and FlhB_C_), and a cytoplasmic ATPase ring complex consisting of FliH, FliI, and FliJ^3^. The FlhA_C_–FlhB_C_-docking platform provides binding sites for the cytoplasmic ATPase complex, flagellar export chaperones (FlgN, FliS, FliT), and export substrates to mediate hierarchical protein targeting and secretion^4^.

**Fig. 1:**
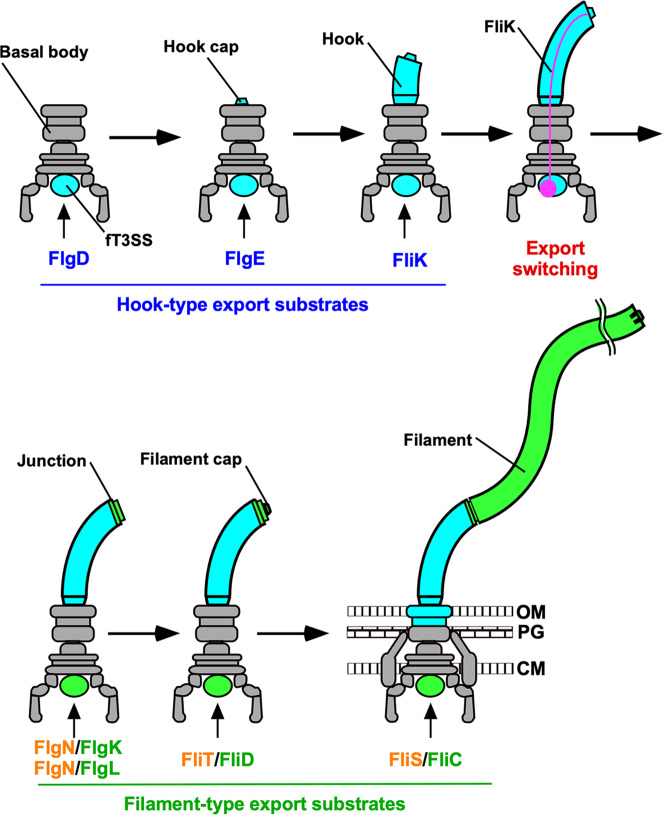
Flagellar assembly pathway. The *Salmonella* flagellum is composed of the basal body, the hook, the hook–filament junction, the filament and the filament cap. Upon completion of basal body assembly, newly exported FlgE molecules polymerize into the hook structure with the help of the hook cap made of FlgD. When the hook reaches its mature length of about 55 nm, the hook cap is replaced by FlgK. FlgK and FlgL self-assemble at the hook tip in this order to form the junction structure. Then, FliD forms the filament cap at the tip of the junction and promotes the assembly of FliC into the filament. A type III protein export apparatus (fT3SS) is located at the flagellar base and transports flagellar building blocks from the cytoplasm to the distal end of the growing flagellar structure. The fT3SS sometimes secretes the FliK ruler to measure the hook length during hook assembly. When the hook reaches its mature length of about 55 nm, the fT3SS switches its substrate specificity, thereby terminating the export of hook-type proteins (FlgD, FlgE, and FliK) and initiating the export of filament-type proteins (FlgK, FlgL, FliD, and FliC). FlgN, FliT, and FliS act as flagellar type III export chaperones specific for FlgK and FlgL, FliD and FliC, respectively. OM outer membrane, PG peptidoglycan layer, CM cytoplasmic membrane.

Flagellar assembly begins with the basal body, followed by the hook (FlgE) with the help of the hook cap (FlgD). After completion of hook–basal body (HBB) assembly, the FlgD cap is replaced by FlgK, and then FlgK and FlgL form the hook–filament junction structure at the hook tip, followed by the assembly of the filament cap (FliD). Finally, newly transported flagellin molecules (FliC) assemble into the filament with the help of the filament cap (Fig. 1)^5^. Flagellar building blocks are classified into two export classes: one is the rod-type (FliE, FlgB, FlgC, FlgF, FlgG, FlgJ) and hook-type class (FlgD, FlgE, and FliK) needed for the assembly of the rod and hook, and the other is the filament-type class (FlgK, FlgL, FlgM, FliC, and FliD) responsible for filament assembly at the hook tip^6,7^. The FlhA_C_–FlhB_C_-docking platform serves as an export switch to induce substrate specificity switching from rod-/hook-type proteins to filament-type ones when the hook reaches its mature length of about 55 nm in *Salmonella*, thereby terminating hook assembly and initiating filament formation (Fig. 1)^8–11^.

The fT3SS uses a secreted molecular ruler protein (FliK) to measure the hook length during hook assembly^4^. FliK is a hook-type protein secreted via the fT3SS during HBB assembly^12^. FliK not only measures the hook length^13–15^ but also switches substrate specificity of the FlhA_C_–FlhB_C_-docking platform (Fig. 1)^11,16,17^. This has been recently verified by in vitro reconstitution experiments using inverted membrane vesicles^18,19^. The N-terminal domain of FliK (FliK_N_) acts as a secreted molecular ruler to measure the hook length^13–15^. When the hook length reaches about 55 nm, a flexible linker region of FliK connecting FliK_N_ and the C-terminal domain (FliK_C_) promotes a conformational rearrangement of FliK_C_, allowing FliK_C_ to interact with FlhB_C_ to terminate the export of the rod-type and hook-type proteins^20,21^.

**Fig. 2:**
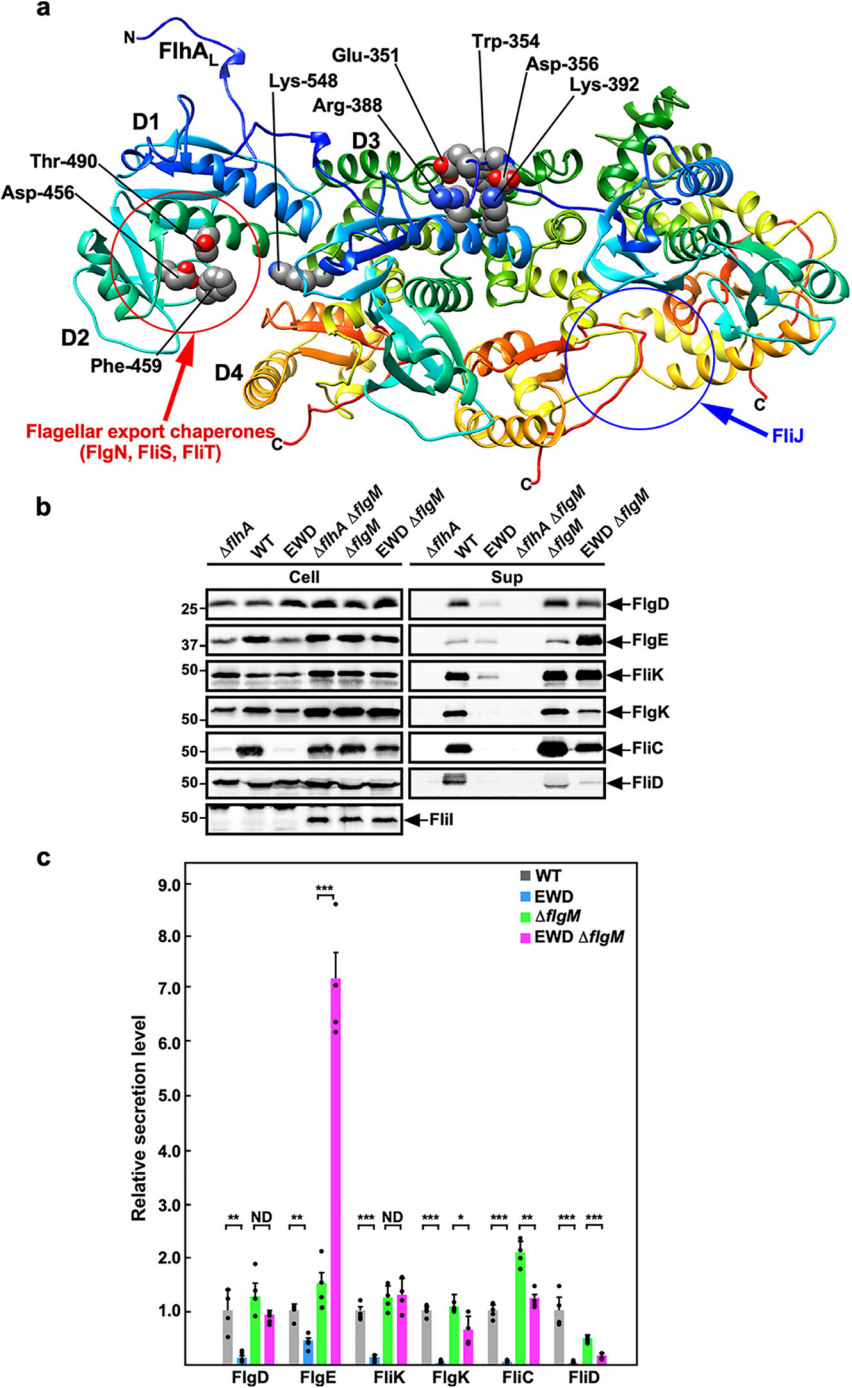
Effect of the *flhA*_*EWD*_ mutation on flagellar protein export. **a** Structural model of the FlhA_C_ ring. Only three FlhA_C_ subunits in the FlhA_C_ nonameric ring model are shown. FlhA_C_ (PDB ID: 3A5I) consists of four domains, D1, D2, D3, and D4 and a flexible linker (FlhA_L_). Glu-351, Trp-354, and Asp-356 of FlhA_L_ binds to the D1 and D3 domains of its neighboring subunit. A well-conserved hydrophobic dimple including Asp-456, Phe-459, and Thr-490 is responsible for the interaction of FlhA_C_ with flagellar export chaperones in complex with filament-type substrates. Phe-459 and Lys-548 are exposed to solvent on the molecular surface when FlhA_C_ adopts the open conformation. FliJ binds not only to FlhA_L_ but also to a large cleft between the D4 domains. **b** Immunoblotting, using polyclonal anti-FlgD (1st row), anti-FlgE (2nd row), anti-FliK (3rd row), anti-FlgK (4th row), anti-FliC (5th row), anti-FliD (6th row), or anti-FliI (7th row) antibody of whole-cell proteins and culture supernatant fractions prepared from the *Salmonella* NH001 strain transformed with pTrc99AFF4 (∆*flhA*), pMM130 (WT), or pYI003 (EWD) and the NH001gM strain transformed with pTrc99FF4A (∆*flhA* ∆*flgM*), pMM130 (∆*flgM*), or pYI003 (EWD ∆*flgM*). The positions of molecular mass markers are indicated on the left. The regions of interest were cropped from original immunoblots shown in Supplementary Fig. 7. **c** Relative secretion levels of flagellar proteins. These data are the average of four independent experiments. The average density of each flagellar protein seen in the culture supernatant derived from wild-type cells was set to 1.0, and then relative band density was calculated. Vertical bars indicate standard deviations. Dots indicate individual data points. The source data are shown in Supplementary Data File. Comparisons between datasets were performed using a two-tailed Student’s *t*-test. A *P* value of <0.05 was considered to be statistically significant difference. *P < 0.05; *P < 0.01; **P < 0.001; ND no statistical difference.

FlhA_C_ (residues 328–692) consists of four domains, D1, D2, D3, and D4, and a flexible linker (FlhA_L_) (residues 328–361) connecting FlhA_C_ with the N-terminal transmembrane domain of FlhA (Fig. 2a)^22^. FlhA_C_ forms a homo-nonamer ring in the fT3SS^23^ and provides binding sites for flagellar export chaperons (FlgN, FliS, and FliT) in complex with their cognate filament-type proteins (Fig. 2a)^24–27^. The flagellar chaperones promote the docking of their cognate filament-type substrates to the FlhA_C_ ring structure to facilitate subsequent unfolding and translocation of the substrates^28,29^. High-speed atomic force microscopy combined with mutational analysis has shown that FlhA_L_ is required for highly cooperative FlhA_C_ ring formation on mica surface^10^. Glu-351, Trp-354, and Asp-356 of FlhA_L_ bind to the D1 and D3 domains of its neighboring FlhA_C_ subunit to stabilize FlhA_C_ ring structure (Fig. 2a)^10^, and the W354A, E351A/D356A, and E351A/W354A/D356A mutations in FlhA_L_ not only inhibit FlhA_C_ ring formation but also reduce the binding affinity of FlhA_C_ for flagellar chaperones in complex with their cognate filament-type substrates, thereby inhibiting the initiation of filament assembly^10^. Therefore, the FliK_C_–FlhB_C_ interaction is postulated to modify the binding mode of FlhA_L_ to its nearest subunit in the FlhA_C_ ring structure upon completion of the hook structure, thereby allowing the flagellar chaperones to bind to FlhA_C_ to initiate the export of filament-type proteins^10,11,30^. However, it remains unknown how FlhA_L_ regulates the interactions of FlhA_C_ with the chaperones during HBB assembly.

In the present study, to clarify the role of FlhA_L_ in the export switching mechanism of fT3SS, we analyzed the interaction between FlhA_L_ and FlhA_C_ and provide evidence suggesting that the interaction of FlhA_L_ with the chaperone-binding site of FlhA_C_ brings the order to flagellar protein export in parallel with the assembly order of the flagellar structure.

## Results

### Isolation of pseudorevertants from the *flhA(E351A/W354A/D356A)* mutant

Glu-351, Trp-354, and Asp-356 of FlhA_L_ bind to the D1 and D3 domains of its neighboring FlhA_C_ subunit to stabilize FlhA_C_ ring structure (Fig. 2a)^10^. The *flhA(E351A/D356A)* (hereafter referred to as *flhA*_*ED*_) and *flhA(W354A)* (hereafter referred to as *flhA*_*W*_) mutants produce the HBBs without the filament attached^10^. Hook lengths of the *flhA*_*ED*_ and *flhA*_*W*_ mutants are 54.0 ± 22.3 nm [mean ± standard deviation (SD)] and 52.9 ± 19.9 nm, respectively, where their SD values are larger than the wild-type one (51.0 ± 6.9 nm), indicating their hook length is not controlled properly^10^. Pull-down assays by GST affinity chromatography have revealed that the *flhA*_*ED*_ and *flhA*_*W*_ mutations reduce the binding affinity of FlhA_C_ for flagellar chaperones in complex with their cognate filament-type substrates^10^. These previous results suggest that the observed interaction between FlhA_L_ and the D1 and D3 domains of its neighboring FlhA_C_ subunit is responsible for making the chaperone-binding site of FlhA_C_ open to allow the chaperones to bind to FlhA_C_ to facilitate the export of filament-type proteins^10^. However, the *flhA(E351A/W354A/D356A)* (hereafter referred to as *flhA*_*EWD*_) mutant does not produce the HBBs^10^, and this raises a question as to why the *flhA*_*EWD*_ mutation inhibits HBB assembly.

To address this question, we first carried out quantitative immunoblotting to measure the amount of flagellar building blocks secreted by the fT3SS. The *flhA*_*EWD*_ mutation significantly reduced the secretion levels of both hook-type (FlgD, FlgE, FliK) and filament-type substrates (FlgK, FliC, FliD) (Fig. 2b, c), indicating that the *flhA*_*EWD*_ mutation significantly reduces the protein transport activity of the fT3SS.

To clarify why and how the *flhA*_*EWD*_ mutation inhibits flagellar protein export, we isolated 14 pseudorevertants from the *flhA*_*EWD*_ mutant. Motility of the pseudorevertants was somewhat better than that of the *flhA*_*EWD*_ mutant but was much poorer than that of wild-type cells (Supplementary Fig. 1a). Export substrates such as FlgD, FlgE, FlgK, and FliD were detected in the culture supernatants of these pseudorevertants (Supplementary Fig. 1b). Consistently, these pseudorevertants produced a couple of flagella on the cell surface (Supplementary Fig. 1c). DNA sequencing revealed that all suppressor mutations are located in the *flgMN* operon. One was the M1I mutation at the start codon of the *flgM* gene (isolated twice), presumably inhibiting FlgM translation. Two suppressor mutations produced a stop codon at position of Gln-52 or Ser-85 of FlgM, resulting in truncation of the C-terminal region of FlgM. Nine suppressor mutations were large deletions in *flgM*. We also found that there was a large deletion in the *flgM* and *flgN* genes, thereby disrupting both FlgM and FlgN. A loss-of-function of FlgM results in a considerable increment in the transcription levels of flagellar genes^31^. Consistently, the cellular levels of flagellar building blocks and the FliI ATPase were higher in the pseudorevertants than those in its parental strain (Fig. 2b).

The interaction of FliJ with FlhA_L_ is required for activation of the fT3SS, and FliH and FliI are required for efficient interaction between FliJ and FlhA_L_^32^. It has been reported that overexpression of export substrates and FliJ by FlgM deletion overcomes the loss of both FliH and FliI to a considerable degree^33^. Because the *flhA*_*EWD*_ mutation reduces the binding affinity of FlhA_C_ for FliJ^10^, this suggests that these *flgM* mutations increase the cytoplasmic levels of FliH, FliI, FliJ, and export substrates to allow the *flhA*_*EWD*_ mutant to export flagellar building blocks for producing a small number of flagella on the cell surface. Therefore, we propose that Glu-351, Trp-354, and Asp-356 of FlhA_L_ also play an important role in the activation mechanism of the fT3SS.

### Effect of deletion of FlhA_L_ on the interaction between FlhA_C_ and FliJ

**Fig. 3:**
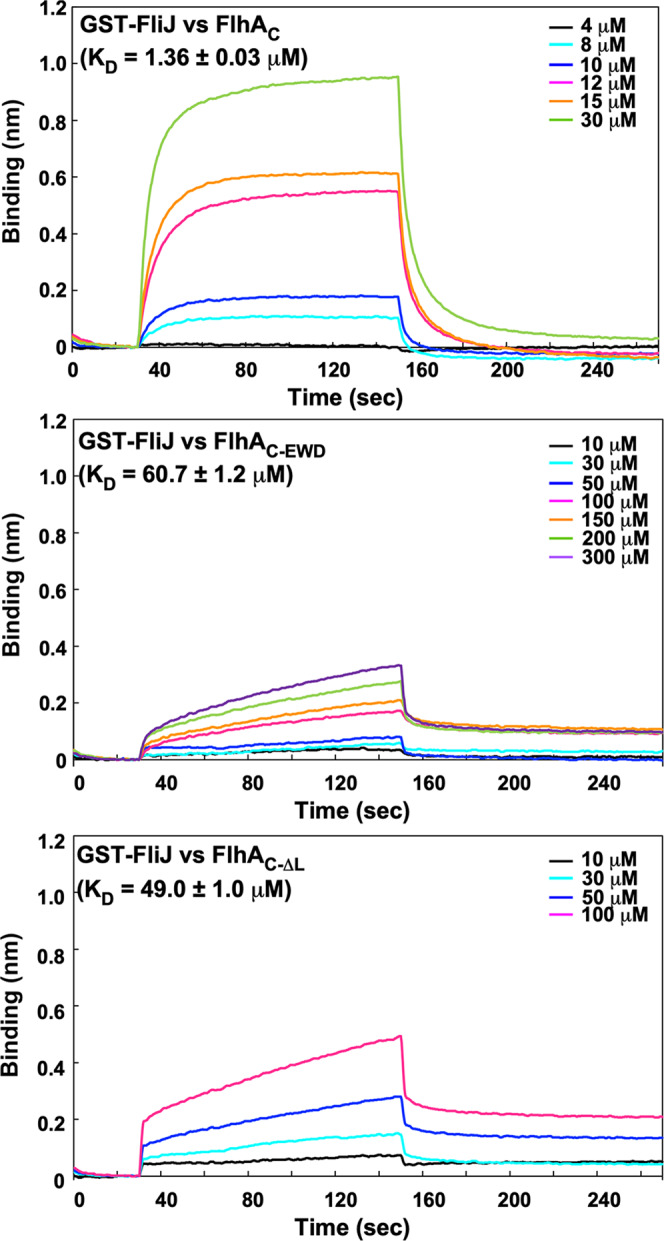
Effect of FlhA linker mutations on the interaction of FlhA_C_ with FliJ. BLI profiles were obtained from the FlhA_C_–FliJ interaction (1st row), the FlhA_C-EWD_–FliJ interaction (2nd row), and the FlhA_C-ΔL_–FliJ interaction (3rd row). GST-FliJ was immobilized to an anti-GST sensor tip. The sensor tip was then dipped into FlhA_C_, FlhA_C-EWD_, or FlhA_C-ΔL_ of various concentrations to measure association before being dipped into the kinetic buffer to measure dissociation. Three independent measurements were carried out. All experiments were performed at 25 °C.

The crystal structure of a FliJ homolog, CdsO, in complex with CdsV_C_, which is a FlhA_C_ homolog, has shown that CdsO binds to a large cleft between domains D4 of neighboring CdsV_C_ subunits in the CdsV_C_ ring structure but not to the linker region of CdsV_C_^34^ (Fig. 2a). To confirm the importance of FlhA_L_ in the interaction between FlhA_C_ and FliJ, we analyzed the binding of FlhA_C_ to immobilized GST-FliJ by Bio-layer interferometry (BLI) measurements^35^. The FliJ–FlhA_C_ interaction showed a complex binding profile (Fig. 3, 1st row) and did not fit the global one-state association-then-dissociation model. Assuming that FlhA_C_ binds to GST-FliJ to form a GST-FliJ/FlhA_C_ complex, followed by a conformational change of this complex, the BLI data fitted well with a two-state reaction model and provided a *K*_D_ value of 1.36 ± 0.03 μM (mean ± SD, *n* = 3). Unlike wild-type FlhA_C_, the association and dissociation processes of FlhA_C_ with the *flhA*_*EWD*_ mutation (FlhA_C-EWD_) or FlhA_C_ lacking FlhA_L_ (FlhA_C-ΔL_) were observed only at protein concentrations above 10 μM (Fig. 3, 2nd and 3rd rows). Their association and dissociation processes were also different from those of wild-type FlhA_C_. The association profile of these mutant proteins was composed of two distinct (fast-on and slow-on) processes, and the dissociation profile was also composed of two distinct (fast-off and slow-off) processes. It has been shown that wild-type FlhA_C_ forms dimer in a protein concentration-dependent manner and that FlhA_L_ is required for efficient dimerization of FlhA_C_^27^. So, their BLI data were fitted well with curves predicted by the Hill equation, with *K*_D_ values of 60.7 ± 1.2 μM (*n* = 3) and 49.0 ± 1.0 μM (*n* = 3) for the FliJ–FlhA_C-EWD_ and FliJ–FlhA_C-ΔL_ interactions, respectively. Thus, both *flhA*_*EWD*_ mutation and deletion of FlhA_L_ reduced the binding affinity of FlhA_C_ for FliJ. Therefore, we conclude that FlhA_L_ is required for the stable interaction between FliJ and FlhA_C_.

### Effect of the *flhA*_*EWD*_ mutation on flagellar protein export by fT3SS in the absence of FlgM

To quantify the amount of flagellar building blocks secreted by the *flhA*_*EWD*_ Δ*flgM* strain, we introduced the Δ*flgM*::*km* allele to the *Salmonella* NH001 (Δ*flhA)* strain to produce the Δ*flgM* and *flhA*_*EWD*_ Δ*flgM* cells. The Δ*flgM*::*km* allele restored motility of the *flhA*_*EWD*_ mutant in a way similar to other *flgM* suppressor mutations (Supplementary Fig. 2a, b). The amount of FlgE secreted by the *flhA*_*EWD*_ Δ*flgM* strain was about fivefold higher than that by the Δ*flgM* strain (Fig. 2b, c), suggesting that this triple mutation significantly increases the binding affinity of the fT3SS for FlgE. However, the *flhA*_*EWD*_ mutation did not affect the levels of FlgD and FliK secretion (Fig. 2b, c). These observations suggest that FlhA_L_ may regulate substrate recognition of the fT3SS for hierarchical protein targeting and secretion among the hook-type substrates. The amount of FlgK, FliC, and FliD secreted by the *flhA*_*EWD*_ Δ*flgM* strain was significantly lower than that by the Δ*flgM* strain (Fig. 2b, c), indicating that the *flhA*_*EWD*_ mutation also affects export switching of the fT3SS from hook-type substrates to filament-type ones.

**Fig. 4:**
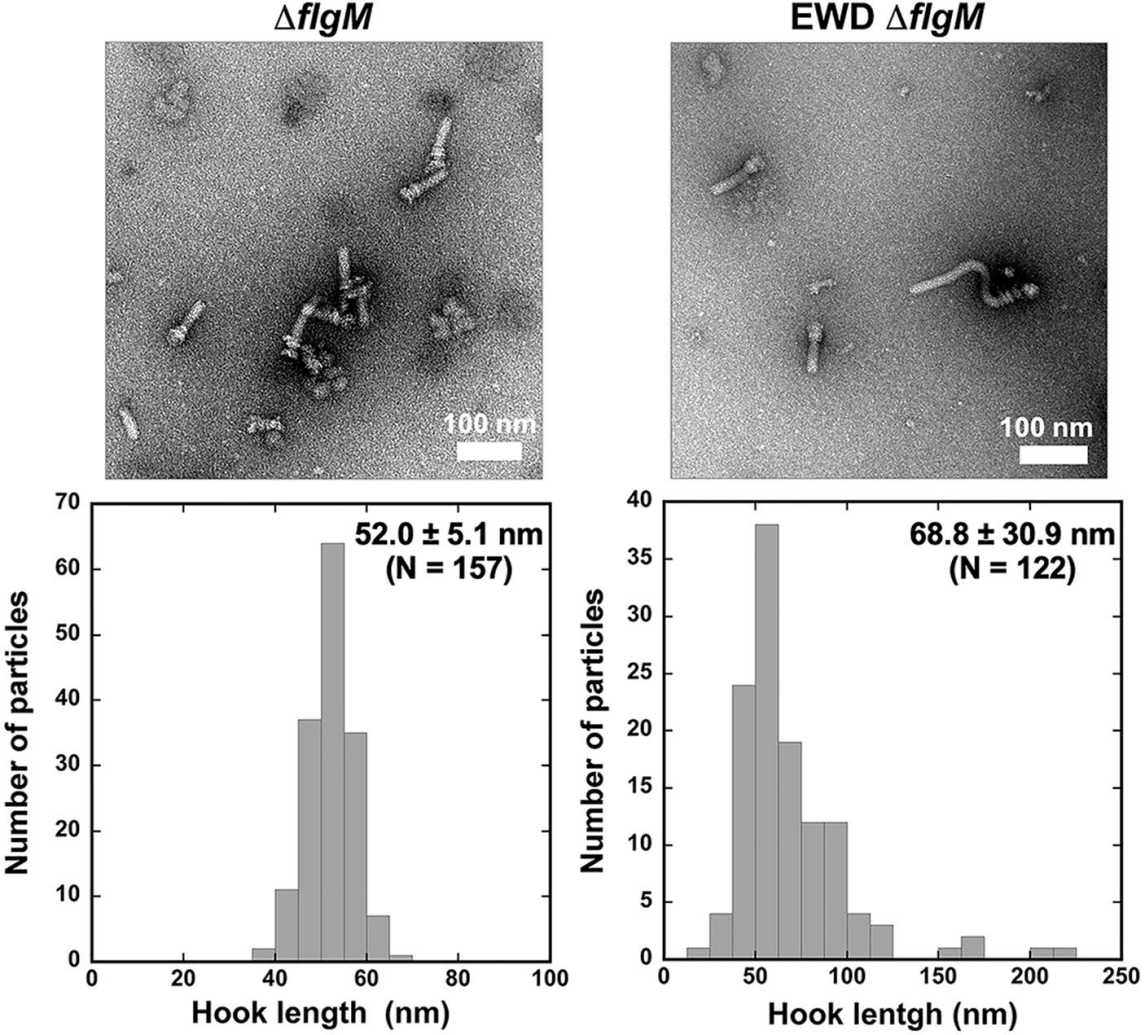
Effect of the *flhA*_*EWD*_ mutation on hook length. Electron micrographs of HBBs and histograms of hook length distribution of NH001gM carrying pMM130 (∆*flgM*) or pYI003 (EWD ∆*flgM*).

We found that the *flhA*_*EWD*_ Δ*flgM* strain secreted a much larger amount of FlgE into the culture media than the Δ*flgM* strain, raising the possibility that the length of the hook produced by this mutant may be longer than the wild-type length. To clarify this, we isolated flagella from the Δ*flgM* and *flhA*_*EWD*_ Δ*flgM* cells and measured their hook length. The hook length of the Δ*flgM* strain was 52.0 ± 5.1 nm (mean ± SD, *n* = 157) (Fig. 4, left panels), which is nearly the same as that of the wild-type strain (51.0 ± 6.9 nm)^10^. This indicates that the loss-of-function mutation of FlgM does not affect the hook length control. In contrast, the average hook length of the *flhA*_*EWD*_ Δ*flgM* strain was 68.8 ± 30.9 nm (*n* = 122) (Fig. 4, right panels), indicating that the hook length control becomes worse in the presence of the *flhA*_*EWD*_ mutation. These suggest that this mutation affects not only the initiation of filament-type protein export but also the termination of hook-type protein export. Because high-speed atomic force microscopy has shown that the *flhA*_*EWD*_ mutation also inhibits highly cooperative FlhA_C_ ring formation^10^, we propose that FlhA_L_ regulates the conformational rearrangement of FlhA_C_ in the ring, which is required for efficient termination of hook assembly and efficient initiation of filament formation at the hook tip.

### Effect of FlhA linker mutations on the hydrodynamic properties of FlhA_C_ in solution

**Fig. 5:**
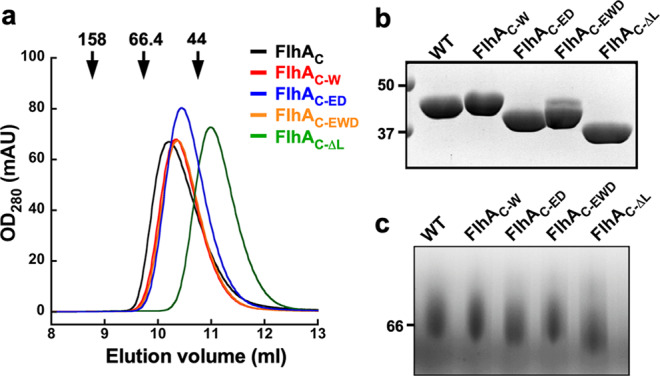
Effect of FlhA linker mutations on the FlhA_C_ conformation. **a** Hydrodynamic properties of FlhA_C_ and its FlhA linker mutant variants. Purified protein samples (10 μM) were run on a Superdex 75HR 10/30 column equilibrated with 50 mM Tri-HCl, pH 8.0, 150 mM NaCl. The elution peaks of His-FlhA_C_ (WT, black), His-FlhA_C-W_ (red), His-FlhA_C-ED_ (blue), His-FlhA_C-EWD_ (orange), and His-FlhA_C-ΔL_ (green) are 10.2, 10.3, 10.5, 10.4, and 11.0 ml, respectively. Arrow indicates the elution peaks of γ-globulin (158 kDa), bovine serum albumin (66.4 kDa), and ovalbumin (43 kDa), which are 8.7, 9.7, and 10.7 ml, respectively. **b** CBB-stained SDS-PAGE gel of purified wild-type FlhA_C_ and its mutant variants. The regions of interest were cropped from an original CBB-stained gel shown in Supplementary Fig. 8a. **c** Blue Native PAGE gel of purified wild-type FlhA_C_ and its mutant variants. The regions of interest were cropped from an original Blue Native PAGE gel shown in Supplementary Fig. 8b.

A well-conserved hydrophobic dimple of FlhA_C_ containing Asp-456, Phe-459, and Thr-490 residues is located at the interface between domains D1 and D2 and is involved in the interactions with the FlgN, FliS, and FliT chaperones in complex with their cognate filament-type substrates (Fig. 2a)^25–27^. The *flhA*_*W*_, *flhA*_*ED*_, and *flhA*_*EWD*_ mutations reduce the binding affinity of FlhA_C_ for these chaperone/substrate complexes^10^. Interestingly, the *flhA(D456V)*, *flhA(F459A),* and *flhA(T490M)* mutations increase the secretion levels of FlgE and FliK by the Δ*fliH-fliI flhB(P28T)* mutant^36^. We found that the *flhA*_*EWD*_ mutation increases the secretion level of FlgE by about fivefold, raising the possibility that FlhA_L_ carrying either of *flhA* linker mutations binds to the hydrophobic dimple of FlhA_C_ not only to facilitate the export of FlgE but also to block the FlhA_C_–chaperone interaction. If this is the case, FlhA_C_ with these mutations would show distinct hydrodynamic properties compared with wild-type FlhA_C_. To clarify this possibility, we performed size exclusion chromatography (SEC) with a Superdex 75 column HR 10/30 column. Wild-type His-FlhA_C_ appeared as a single peak at an elution volume of 10.2 ml, which corresponds to the deduced molecular mass of His-FlhA_C_ (about 43 kDa) (Fig. 5a). His-FlhA_C_ with the *flhA*_*W*_ (FlhA_C-W_), *flhA*_*ED*_ (FlhA_C-ED_) or *flhA*_*EWD*_ mutation (FlhA_C-EWD_) and FlhA_C-ΔL_ lacking FlhA_L_ appeared as a single peak at an elution volume of 10.3, 10.5, 10.4, and 11.0 ml, respectively (Fig. 5a), indicating that these mutant variants exist as a monomer in solution. FlhA_C-ED_ exhibited a delayed elution behavior compared with the wild type. Furthermore, FlhA_C-ED_ showed a slightly faster mobility in both sodium dodecyl sulfate polyacrylamide gel electrophoresis (SDS-PAGE) and native PAGE gels (Fig. 5b, c). Far-UV CD measurements revealed that the *flhA*_*ED*_ mutation did not affect the secondary structures of FlhA_C_ (Supplementary Fig. 3). These suggest that FlhA_C-ED_ adopts a more compact conformation than wild-type FlhA_C_. The elution peak position of FlhA_C-EWD_ was between those of the wild type and FlhA_C-ED_ (Fig. 5a). Because FlhA_C-EWD_ showed two different bands on SDS-PAGE gels, with a slower mobility band corresponding to wild-type FlhA_C_ and a faster one corresponding to FlhA_C-ED_ (Fig. 5b), we suggest that FlhA_C-EWD_ exists in an equilibrium between the wild-type conformation and the compact conformation. Therefore, we suggest that the *flhA*_*ED*_ mutation is required to make FlhA_C_ more compact.

### Effect of FlhA linker mutations on methoxypolyethylene glycol 5000 maleimide (mPEG-maleimide) modifications of Cys-459 and Cys-548

FlhA_C_ structures adopt open, semi-closed, and closed confromations^22–24,27,30,37^. A large open cleft between domains D2 and D4 is seen in the open form, but not in the closed form. As a result, Phe-459 and Lys-548, which are both located in the cleft between domains D2 and D4, are fully exposed to solvent on the molecular surface of the open conformation of FlhA_C_ but are in close proximity to each other in the closed conformation^22,30,37^. To test whether mutations in FlhA_L_ bias FlhA_C_ towards the closed structure, we performed Cys modification experiments with mPEG-maleimide. FlhA_C_ with the F459C/K548C substitutions modified by mPEG-maleimide showed much slower mobility shift (Supplementary Fig. 4, left panel), in agreement with a previous report^30^. The *flhA*_*W*_, *flhA*_*ED*_*,* and *flhA*_*EWD*_ mutations did not inhibit Cys modifications with mPEG-maleimide (Supplementary Fig. 4, right panel), indicating that FlhA_C_ with these mutations does not adopt the closed conformation.

### Crystal structure of FlhA_C-ED_

**Fig. 6:**
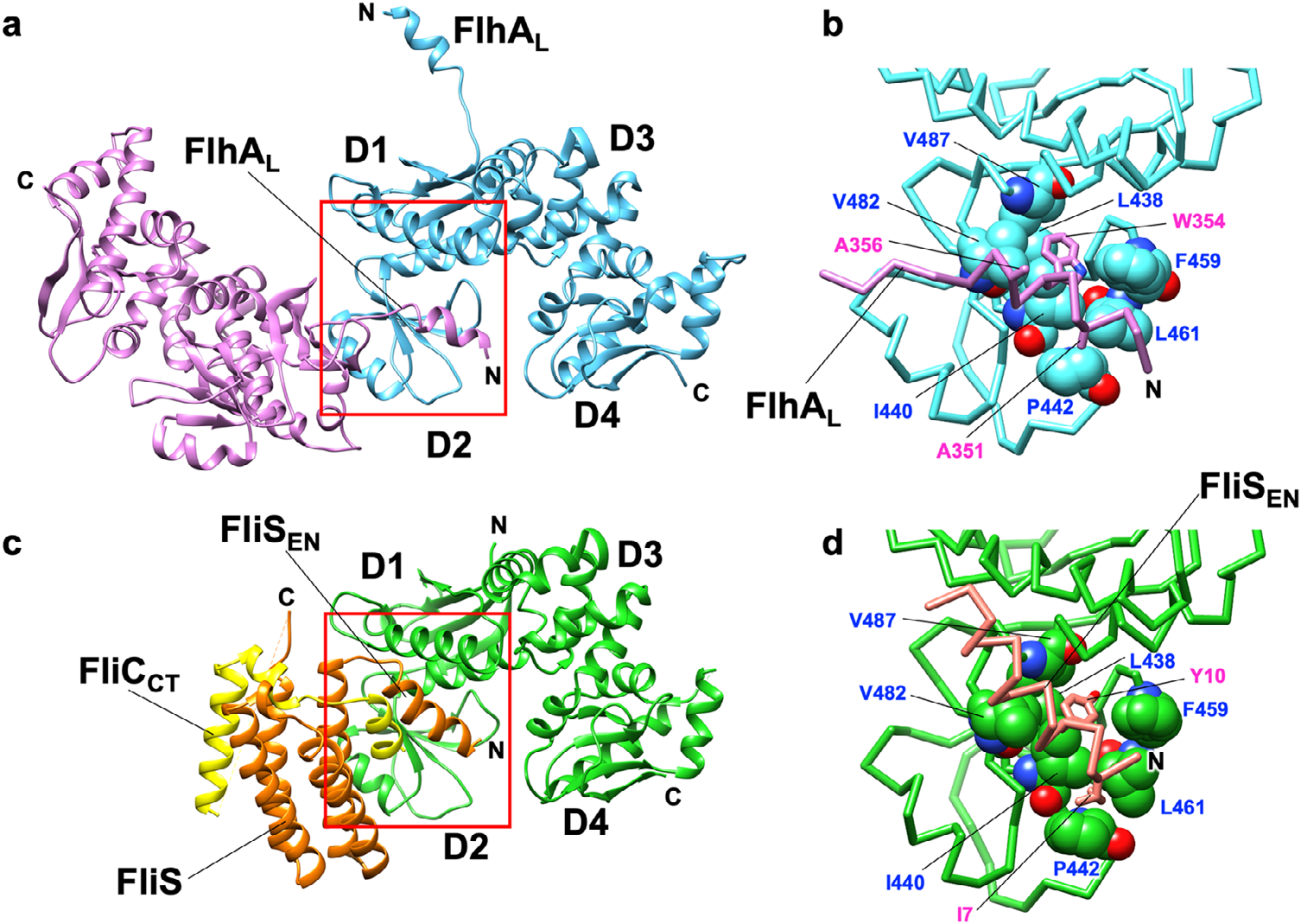
Interaction between FlhA_L_ and a well-conserved hydrophobic dimple of its neighboring FlhA_C_ in the crystal of FlhA_C-ED_. **a** FlhA_L_ of Mol-A (magenta) interacts with neighboring Mol-A (cyan) related by a crystallographic symmetry. **b** Close-up view of the interaction between FlhA_L_ and the hydrophobic dimple shown by a red box in **a**. Residues that form the hydrophobic dimple are indicated by balls. The side chains of Ala-351, Trp-354, and Ala-356 in FlhA_L_ are shown in stick models. **c** Interaction between FlhA_C_ (green) and FliS (orange) fused with the C-terminal region of FliC (yellow) (PDB code: 6CH3). **d** Close-up view of the interaction between the extreme N-terminal region of FliS (FliS_EN_) and the hydrophobic dimple shown by a red box in **c**. The residues that form the hydrophobic dimple are indicated by ball. The side chains of Ile-7 and Tyr-10 of FliS_EN_ are shown in stick models.

To investigate whether FlhA_L_ binds to the hydrophobic dimple of FlhA_C_ to make FlhA_C-ED_ more compact, we explored crystallization conditions of FlhA_C-ED_ for a molecular packing distinct from the open (PDB code: 3A5I)^22^ and semi-closed (PDB code: 6AI0)^30^ forms of wild-type FlhA_C_. We found a new orthorhombic crystal that diffracted up to 2.8 Å resolution, with unit cell dimensions *a* = 71.7 Å, *b* = 96.2 Å, *c* = 114.1 Å (Table 1) and the asymmetric unit containing two FlhA_C_ molecules (A and B). Mol-A adopts an open conformation similar to the 3A5I structure (Supplementary Fig. 5a, c) whereas Mol-B shows a semi-closed conformation similar to the 6AI0 structure (Supplementary Fig. 5b, d). The residues from Val-349 to Val-357 in FlhA_L_ of Mol-A form an α-helix, which interacts with the hydrophobic dimple of a neighboring Mol-A molecule related by a crystallographic symmetry (Fig. 6a). Trp-354 fits into the hydrophobic dimple, and Ala-351 hydrophobically contacts with Pro-442 on the periphery of the dimple (Fig. 6b and Supplementary Fig. 6a). These interactions resemble the interaction between the N-terminal α-helix of FliS and the hydrophobic dimple of FlhA_C_ (PDB ID: 6CH3)^27^ (Fig. 6c). Ile-7 and Tyr-10 of the N-terminal α-helix of FliS is in the corresponding position of Ala-351 and Trp-354 of FlhA_L_, respectively. Tyr-10 fits into the hydrophobic dimple of FlhA_C_, and Ile-7 interacts with Pro-442 of FlhA_C_ (Fig. 6d). These observations suggest that FlhA_L_ and flagellar chaperones bind competitively to a common binding site on FlhA_C_ and that the dissociation of FlhA_L_ from this binding site is required for the binding of the flagellar chaperones to FlhA_C_. When Ala-351 and Ala-356 of FlhA_C-ED_ in the crystal structure were replaced back to the original Glu-351 and Asp-356 residues, respectively, the side chain arm of Glu-351 can form a hydrophobic contact with Pro-442 (Supplementary Fig. 6b), suggesting that FlhA_L_ can bind to the hydrophobic dimple of FlhA_C_ even in the wild type without the *flhA*_*ED*_ mutation. Because the introduced Ala residues would increase the helical propensity of residues 349–357 of FlhA_L_ as seen in the crystal, we suggest that the *flhA*_*ED*_ mutation allowed residues 349–357 of FlhA_L_ to efficiently form an α-helix to stabilize the binding of FlhA_L_ to the hydrophobic dimple of FlhA_C_.

**Table 1:** Data collection and refinement statistics.

	FlhA_C_(E351A/D356A)
**Data collection**	
Space group	*P*2_1_2_1_2_1_
Cell dimensions	
* a*, *b*, *c* (Å)	71.7, 96.2, 114.1
*α*, *β*, *γ* (°)	90.0, 90.0, 90.0
Resolution (Å)	73.5–2.80 (2.95–2.80)^a^
*R*_merge_	0.074 (0.317)
CC(1/2)	0.995 (0.915)
*I* / *σI*	8.1 (2.8)
Completeness (%)	97.1 (94.6)
Redundancy	3.4 (3.1)
**Refinement**	
Resolution (Å)	73.5–2.80 (2.87–2.80)
No. of reflections	19,301 (1294)
*R*_work_/*R*_free_	23.2/29.0 (33.5/42.1)
No. of atoms	
Protein	5252
Ligand/ion	0
Water	0
*B*-factors	
Protein	70.0
Ligand/ion	–
Water	–
R.m.s. deviations	
Bond lengths (Å)	0.003
Bond angles (°)	0.680

Number of crystals: 1.

^a^Values in parentheses are for highest-resolution shell.

### Effect of FlhA linker mutations on the interaction of FlhA_C_ with the FlgN chaperone

**Fig. 7:**
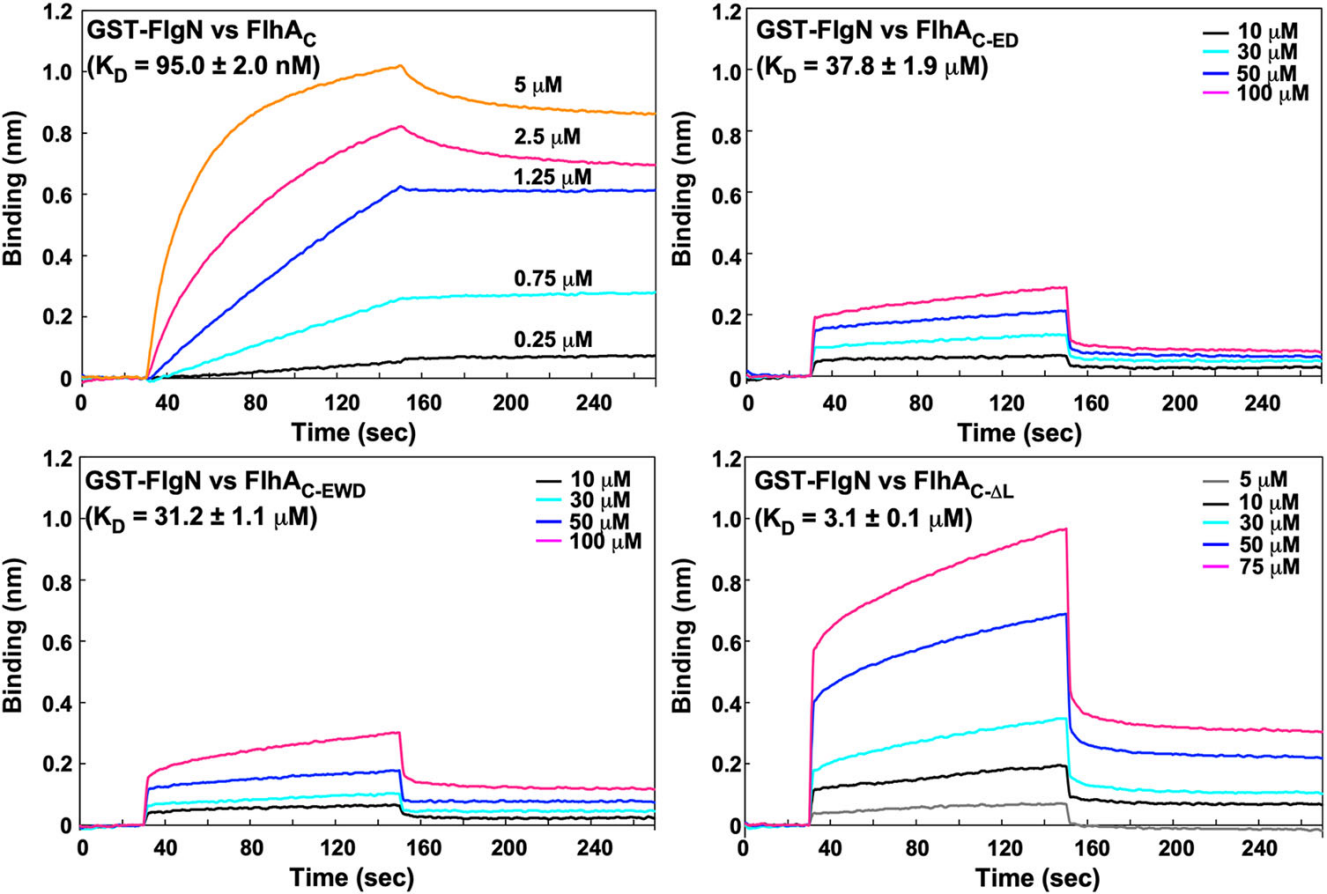
Effect of FlhA linker mutations on the interaction of FlhA_C_ with FlgN. BLI profiles were obtained from the FlhA_C_–FlgN interaction (upper, left panel), the FlhA_C-ED_–FlgN interaction (upper, right panel), the FlhA_C-EWD_–FlgN interaction (lower, left panel) and the FlhA_C-ΔL_–FlgN interaction (lower, right panel). GST-FlgN was immobilized to an anti-GST sensor tip. The sensor tip was then dipped into FlhA_C_, FlhA_C-ED_, FlhA_C-EWD_, or FlhA_C-ΔL_ of various concentrations to measure association before being dipped into the kinetic buffer to measure dissociation. Three independent measurements were carried out. All experiments were performed at 25 °C.

We found that FlhA_L_ with the *flhA*_*ED*_ mutation binds to the chaperone-binding site in its neighboring subunit in the crystal. If this interaction reflects the functional state of FlhA_C_, the *flhA*_*ED*_ mutation would affect the docking process of FlgN to FlhA_C_. To clarify this hypothesis, we performed BLI measurements. When GST-FlgN was tethered to a sensor chip and then allowed FlhA_C_ of various concentrations to bind to immobilized GST-FlgN, the interaction between FlgN and FlhA_C_ showed a typical BLI profile (Fig. 7). The association and dissociation rate constants were measured to be about 8.23 ± 0.20 × 10^3^ M^−1^ S^−1^ and 7.81 ± 0.03 × 10^−4^ S^−1^, respectively, giving a *K*_D_ value of 95.0 ± 2.0 nM (mean ± SD, *n* = 3). This *K*_D_ value is in agreement with previous data obtained by surface plasmon resonance^24^. Unlike wild-type FlhA_C_, FlhA_C-ED_ and FlhA_C-EWD_ did not bind to immobilized GST-FlgN at protein concentrations less than 10 μM, indicating that FlhA_L_ with either of these two mutations inhibits the binding of FlhA_C_ to FlgN. The association and dissociation processes of FlhA_C-ED_ and FlhA_C-EWD_ were observed with an increase in the protein concentration (Fig. 7). However, these mutations caused fast-on and fast-off binding profiles (Fig. 7). Assuming that GST-FlgN binds to FlhA_C-ED_ or FlhA_C-EWD_ by inducing the dissociation of FlhA_L_ with either of these *flhA* mutations from the chaperone-binding site so that GST-FlgN forms a complex with FlhA_C-ED_ or FlhA_C-EWD_ on the sensor chip, their BLI data fitted well with a two-state reaction model, giving *K*_D_ values of 37.8 ± 1.9 μM (*n* = 3) and 31.2 ± 1.1 μM (*n* = 3) for the FlgN–FlhA_C-ED_ and FlgN–FlhA_C-EWD_ interactions, respectively.

We next investigated whether deletion of FlhA_L_ affect the binding process of FlgN to FlhA_C_. The association and dissociation processes of FlhA_C-ΔL_ were clearly observed at protein concentrations above 5 μM, and the BLI signals for the FlgN–FlhA_C-ΔL_ interaction were much stronger at the same protein concentrations compared to the FlgN–FlhA_C-ED_ and FlgN–FlhA_C-EWD_ interactions (Fig. 7). Furthermore, the association and dissociation profiles of FlhA_C-ΔL_ were different from those of FlhA_C-ED_ and FlhA_C-EWD_. Its BLI data did not fit the global one-state association-then-dissociation model, but fitted with a heterogeneous reaction model, showing a *K*_D_ value of 3.1 ± 0.1 μM (*n* = 3). Thus, the binding affinity of FlhA_C-ΔL_ for FlgN was higher than those of FlhA_C-ED_ and FlhA_C-EWD_. This suggests that FlhA_L_ with either *flhA*_*ED*_ or *flhA*_*EWD*_ mutation inhibits the binding of FlgN to FlhA_C_. Because the binding affinity of FlhA_C-ΔL_ for FlgN was much lower than that of wild-type FlhA_C_, we suggest that FlhA_L_ is required to keep FlhA_C_ in the open form so that FlgN can efficiently and stably bind to the well-conserved hydrophobic dimple of FlhA_C_.

## Discussion

The FlhA_C_ ring serves as the docking platform for flagellar export chaperones in complex with their cognate substrates and facilitates the export of filament-type proteins to form the filament at the hook tip after completion of hook assembly^24–27^. The FlhA_C_ ring also ensures the strict order of flagellar protein export, thereby allowing the huge and complex flagellar structure to be built efficiently on the cell surface^10,11,30,36^. An interaction of FlhA_L_ with its neighboring FlhA_C_ subunit in the nonamer ring is required for the initiation of filament-type protein export upon completion of hook assembly^10^. However, it remained unclear how the FlhA_C_ ring mediates such hierarchical protein export during flagellar assembly.

In this study, we first performed genetic analyses of the *flhA*_*EWD*_ mutant and found that this mutation reduces the protein transport activity of the fT3SS significantly (Fig. 2b, c). We also found that both the *flhA*_*EWD*_ mutation and deletion of FlhA_L_ reduce the binding affinity of FlhA_C_ for FliJ (Fig. 3). Because the interaction between FliJ and FlhA_L_ is required for activation of the fT3SS^32^, we propose that Glu-351, Trp-354, and Asp-356 of FlhA_L_ is required for stable interaction of FlhA_L_ with FliJ to fully activate the fT3SS to facilitate flagellar protein export.

It has been reported that either *flhA(D456V)*, *flhA(F459A),* or *flhA(T490M)* mutation in the flagellar chaperone-binding site in FlhA_C_ increases the levels of FlgE and FliK secretion by the Δ*fliH-fliI flhB(P28T)* mutant^36^, suggesting that this chaperone-binding site is also involved in the export of hook-type substrates. Here, we found that the *flhA*_*EWD*_ mutation significantly increased the secretion level of FlgE by a Δ*flgM* mutant (Fig. 2b, c), thereby producing longer hooks (Fig. 4). This indicates that the *flhA*_*EWD*_ mutation affects the termination of hook-type protein export, suggesting that an interaction between FlhA_L_ and the chaperone-binding site of FlhA_C_ coordinates the export of hook-type proteins with hook assembly in a highly organized and well-controlled manner. Furthermore, we also found that this triple mutation also reduced the secretion levels of filament-type substrates significantly (Fig. 2b, c), thereby reducing the number of flagellar filaments per cell (Supplementary Figs. 1 and 2). Taken all together, we propose that FlhA_L_ serves as a structural switch for substrate specificity switching of the fT3SS from hook type to filament type and that Glu-351, Trp-354, and Asp-356 of FlhA_L_ are directly involved in this export switching mechanism.

**Fig. 8:**
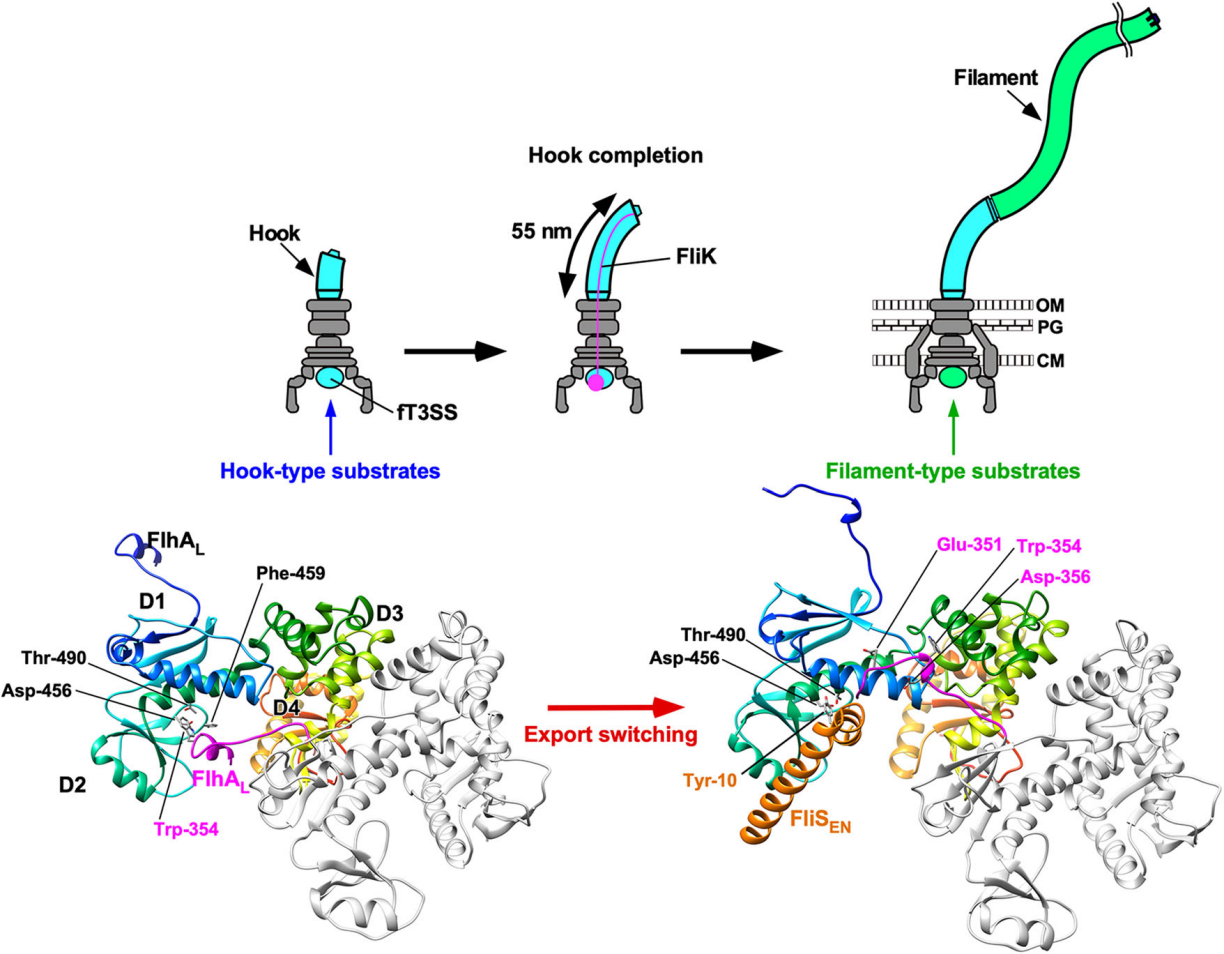
Structural rearrangements of FlhA_L_ responsible for export switching of fT3SS. Trp-354 of FlhA_L_ binds to a well-conserved hydrophobic dimple containing Asp-456, Phe-459, and Thr-490 of its neighboring FlhA_C_ subunit in the FlhA_C_ ring not only to inhibit the interaction of FlhA_C_ with flagellar chaperones in complex with their cognate filament-type substrates but also to facilitate the export of the hook protein during hook assembly. When the hook reaches its mature length of about 55 nm, an interaction between FliK_C_ and FlhB_C_ triggers a conformational rearrangement of the FlhA_C_ ring so that FlhA_L_ dissociates from the hydrophobic dimple and binds to the D1 and D3 domains of the neighboring FlhA_C_ subunit, allowing the chaperones to bind to FlhA_C_ to facilitate the export of their cognate substrates for filament assembly.

It has been reported that the *flhA*_*W*_, *flhA*_*ED*_, and *flhA*_*EWD*_ mutations inhibit interactions between FlhA_C_ and flagellar chaperones in complex with their cognate filament-type substrates^10^, suggesting that FlhA_L_ regulates the binding affinity of FlhA_C_ for flagellar chaperones. The crystal structure of FlhA_C-ED_ we solved in this study showed that FlhA_L_ of a Mol-A molecule bound to the hydrophobic dimple of the flagellar chaperone-binding site of its nearest Mol-A in the crystal (Fig. 6). Although the relative orientations of these Mol-A molecules in the crystal differs from those in the FlhA_C_ nonameric ring, FlhA_L_ should be able to bind to the hydrophobic dimple of FlhA_C_ in the nonamer ring structure as well because of a highly flexible nature of FlhA_L_ (Fig. 8). The C-terminal region of FlhA_L_ is flexible enough to allow such subunit orientations without changing the essential interaction between FlhA_L_ and the chaperone-binding site of FlhA_C_ (Fig. 8), as it has been shown to have various conformations in the known FlhAc structures^28^. BLI measurements indicated that FlhA_L_ with either *flhA*_*ED*_ or *flhA*_*EWD*_ mutation inhibits the docking process of FlgN to FlhA_C_ (Fig. 7). Because we also found that FlhA_L_ is required for stable interaction between FlgN and FlhA_C_ (Fig. 7), we propose that the interaction between FlhA_L_ and the hydrophobic dimple of its neighboring FlhA_C_ subunit suppresses the docking of flagellar chaperones to the FlhA_C_ ring platform during HBB assembly and that the hook assembly completion induces the detachment of FlhA_L_ from the dimple through an interaction between FliK_C_ and FlhB_C_ and its attachment to the D1 and D3 domains to induce structural remodeling of the entire FlhA_C_ ring, thereby terminating hook assembly and initiating filament formation (Fig. 8). Because FlhA_C-ED_ monomer adopts a more compact conformation compared with the wild-type FlhA_C_ monomer as judged by SEC (Fig. 5a), FlhA_L_ may bind to FlhA_C_ in a *cis* manner as well. Therefore, it is also possible that FlhA_L_ may block the docking of the flagellar chaperones to FlhA_C_ by covering the binding site of the same FlhA_C_ molecule.

## Methods

### Bacterial strains, plasmids, transductional crosses, and DNA manipulations

Bacterial strains and plasmids used in this study are listed in Table 2. P22-mediated transductional crosses were performed with P22HT*int*. DNA manipulations were performed using standard protocols^38^. Site-directed mutagenesis were carried out using the QuikChange site-directed mutagenesis method as described in the manufacturer’s instructions (Stratagene). DNA sequencing reactions were carried out using BigDye v3.1 (Applied Biosystems) and then the reaction mixtures were analyzed by a 3130 Genetic Analyzer (Applied Biosystems).

**Table 2:** Strains and plasmids used in this study.

Strain/plasmid	Relevant characteristics	References
*E. coli*		
BL21 Star (DE3)	Overexpression of proteins	Novagen
*Salmonella*		
NH001	∆*flhA*	^47^
NH001gM	∆*flhA ∆flgM*::*km*	This study
YI1003-xx	Pseudorevertants isolated from NH001 carrying pYI003	This study
*Plasmids*		
pTrc99AFF4	Expression vector	^48^
pMM130	pTrc99AFF4/FlhA	^49^
pMMGN101	pGEX-6p-1/GST-FlgN	^25^
pMMJ1002	pGEX-6p-1/GST-FliJ	^50^
pYI003	pTrc99AFF4/FlhA(E351A/W354A/D356A)	^10^
pYI008	pET15b/His-FlhA_C_ (residues 328–692 of FlhA)	^10^
pYI009	pET15b/His-FlhA_C_(W354A)	^10^
pYI010	pET15b/His-FlhA_C_(E351A/D356A)	^10^
pYI012	pET15b/His-FlhA_C_(E351A/W354A/D356A)	^10^
pYI008(F459C)	pET15b/His-FlhA_C_(F459C)	^30^
pYI008(K548C)	pET15b/His-FlhA_C_(K548C)	^30^
pYI008(F459C/K548C)	pET15b/His-FlhA_C_(F459C/K548C)	^30^
pYI009(F459C/K548C)	pET15b/His-FlhA_C_(W354A/F459C/K548C)	This study
pYI010(F459C/K548C)	pET15b/His-FlhA_C_ FlhA_C_(E351A/D356A/F459C/K548C)	This study
pYI012(F459C/K548C)	pET15b/ His-FlhA_C_(E351A/ W354A/D356A/F459C/K548C)	This study
pMKMhA008-1	pET15b/His-FlhA_C_ lacking FlhA_L_ (residues 328–361)	This study

### Motility assays

We transformed *Salmonella enterica* strains NH001 and NH001gM with a pTrc99A-based plasmid encoding wild-type FlhA or its mutant variant. Fresh transformants were inoculated into soft agar plates [1% (w/v) triptone, 0.5% (w/v) NaCl, 0.35% Bacto agar] containing 100 μg ml^−1^ ampicillin and incubated at 30 °C. At least five independent measurements were performed.

### Secretion assays

*S. enterica* cells were grown in T-broth [1% (w/v) tryptone, 0.5% (w/v) NaCl] containing ampicillin at 30 °C with shaking until the cell density had reached an OD_600_ of ca. 1.4–1.6. Cultures were centrifuged to obtain cell pellets and culture supernatants. The cell pellets were resuspended in a sample buffer solution [62.5 mM Tris-HCl, pH 6.8, 2% SDS, 10% glycerol, 0.001% bromophenol blue] containing 1 μl of 2-mercaptoethanol. Proteins in the culture supernatants were precipitated by 10% trichloroacetic acid and suspended in a Tris/SDS loading buffer (one volume of 1 M Tris, nine volumes of 1× sample buffer solution)^39^ containing 1 μl of 2-mercaptoethanol. Both whole cellular proteins and culture supernatants were normalized to a cell density of each culture to give a constant cell number. After boiling proteins in both whole cellular and culture supernatant fractions at 95 °C for 3 min, these protein samples were separated by SDS-PAGE (normally 12.5% acrylamide) and transferred to nitrocellulose membranes (Cytiva) using a transblotting apparatus (Hoefer). Then, immunoblotting with polyclonal anti-FlgD, anti-FlgE, anti-FlgK, anti-FliC, anti-FliD, anti-FliI, or anti-FliK antibody was carried out using iBand Flex Western Device (Thermo Fisher Scientific). Detection was performed with Amersham ECL Prime western blotting detection reagent (Cytiva). Chemiluminescence signals were captured by a Luminoimage analyzer LAS-3000 (GE Healthcare). The band intensity of each blot was analyzed using an image analysis software, CS Analyzer 4 (ATTO, Tokyo, Japan). More than three independent experiments were performed.

### Electron microscopy observation of negatively stained *Salmonella* cells

*S. enterica* cells were exponentially grown in 5 ml L-broth [1% (w/v) tryptone, 0.5% (w/v) yeast extract, 0.5% (w/v) NaCl] containing ampicillin at 30 °C. Five microliters of the cell culture was applied to carbon-coated copper grids and then negatively stained with 0.5% (w/v) phosphotungstic acid, pH 6.5. Micrographs were recorded at a magnification of ×1200 with a JEM-1010 transmission electron microscope (JEOL) operating at 100 kV.

### Observation of flagellar filaments with a fluorescent dye

*S. enterica* cells were grown in T-broth containing ampicillin. The cells were attached to a coverslip (Matsunami glass, Japan), and unattached cells were washed away with motility buffer (10 mM potassium phosphate pH 7.0, 0.1 mM EDTA, 10 mM l-sodium lactate). Then, the flagellar filaments were labeled using anti-FliC antibody and anti-rabbit IgG conjugated with Alexa Fluor 594 (Invitrogen) as described previously^40^. After washing twice with the motility buffer, epi-fluorescence of Alexa Fluor 594 was observed by an inverted fluorescence microscope (IX-83, Olympus) with a ×150 oil immersion objective lens (UApo150XOTIRFM, NA 1.45, Olympus) and an Electron-Multiplying Charge-Coupled Device camera (iXon^EM^ + 897-BI, Andor Technology)^41^. Fluorescence images were analyzed using ImageJ software version 1.52 (National Institutes of Health).

### Bio-layer interferometry

His-FlhA_C_ and its mutant variants were purified by Ni affinity chromatography, followed by SEC as described previously^28^. GST-FliJ and GST-FlgN were purified by GST affinity chromatography as described previously^25,28^. Purified protein samples were dialyzed overnight against a kinetic buffer [PBS (8.8 g of NaCl, 0.2 g of KCl, 3.63 g of Na_2_HPO_4_·12H_2_O, 0.24 g of KH_2_PO_4_, pH 7.4 per liter), 0.1% bovine serum albumin, 0.002% Tween-20] at 4 °C with three changes of PBS.

BLI measurements were carried out using a BLItz (FortéBio). GST-FliJ or GST-FlgN was immobilized to an anti-GST sensor tip (FortéBio). The sensor tip was then dipped into His-FlhA_C_ or its mutant variants to measure association before being dipped into the kinetic buffer to measure dissociation. Data were reference subtracted and fit to various model using BLItz Pro software (FortéBio) and BIAevaluation software (GE Healthcare).

### Hook length measurements

The HBBs were purified from NH004gM carrying pMM130 or pYI003 as described previously^36^. *Salmonella* cells were grown in L-broth containing ampicillin at 30 °C with shaking until the cell density had reached an OD_600_ of ca. 1.0. The cultures were centrifuged (10,000*g*, 10 min, 4 °C), and the cell pellets were suspended in 20 ml of ice-cold 0.1 M Tris-HCl pH 8.0, 0.5 M sucrose, followed by addition of EDTA and lysozyme at the final concentrations of 10 mM and 0.1 mg ml^−1^, respectively. The cell suspensions were stirred for 30 min at 4 °C, and then were solubilized on ice for 1 h by adding Triton X-100 and MgSO_4_ at final concentrations of 1% (w/v) and 10 mM, respectively. The cell lysates were adjusted to pH 10.5 with 5 M NaOH and then centrifuged (10,000*g*, 20 min, 4 °C) to remove cell debris. After ultracentrifugation (45,000*g*, 60 min, 4 °C), pellets were resuspended in 10 mM Tris-HCl, pH 8.0, 5 mM EDTA, 1% Triton X-100 and the solution was loaded a 20–50% (w/w) sucrose density gradient in 10 mM Tris-HCl, pH 8.0, 5 mM EDTA, 1% Triton X-100. After ultracentrifugation (49,100*g*, 13 h, 4 °C), intact flagella were collected and ultracentrifuged (60,000*g*, 60 min, 4 °C). Pellets were suspended in 50 mM glycine, pH 2.5, 0.1% Triton X-100 to depolymerize the flagellar filaments. After ultracentrifugation (60,000*g*, 60 min, 4 °C), pellets were resuspended in 50 μl of 10 mM Tris-HCl, pH 8.0, 5 mM EDTA, 0.1% Triton X-100. The HBBs were negatively stained with 2% (w/v) uranyl acetate. Electron micrographs were recorded with a JEM-1011 transmission electron microscope (JEOL, Tokyo, Japan) operated at 100 kV and equipped with a F415 CCD camera (TVIPS, Gauting, Germany). Hook length was measured by ImageJ version 1.52 (National Institutes of Health).

### Size exclusion chromatography

SEC was performed with a Superdex 75HR 10/30 column (GE Healthcare). Purified His-FlhA_C_ and its mutant variants (10 μM) were run on the SEC column equilibrated with 50 mM Tri-HCl, pH 8.0, 150 mM NaCl at a flow rate of 0.5 ml min^−1^. γ-Globulin (158 kDa), bovine serum albumin (66.4 kDa) and ovalbumin (43 kDa) were used as size markers. All fractions were run on SDS-PAGE and then analyzed by Coomassie Brilliant blue (CBB) staining.

### Native PAGE

Purified His-FlhA_C_ and its mutant variants (14.4 μM) were run on Native PAGE Novex Bis-Tris gels as described in the manufacturer’s instructions (Invitrogen).

### Far-UV CD spectroscopy

Far-UV CD spectroscopy of His-FlhA_C_ or its mutant variants was carried out at room temperature using a Jasco-720 spectropolarimeter (JASCO International Co., Tokyo, Japan) as described previously^42^. The CD spectra of His-FlhA_C_ and its mutant forms were measured in 20 mM Tris-HCl, pH 8.0 using a cylindrical fused quartz cell with a path length of 0.1 cm in a wavelength range of 200–260 nm. Spectra were obtained by averaging five successive accumulations with a wavelength step of 0.5 nm at a rate of 20 nm min^−1^, response time of 8 s, and bandwidth of 2.0 nm.

### Cystein modification by mPEG-maleimide

His-FlhA_C(F459C)_, His-FlhA_C(K548C)_, His-FlhA_C(F459C/K548C)_, His-FlhA_C-W(F459C/K548C)_, His-FlhA_C-ED(F459C/K548C),_ and His-FlhA_C-EWD(F459C/K548C)_ were dialyzed overnight against PBS (8 g of NaCl, 0.2 g of KCl, 3.63 g of Na_2_HPO_4_ 12H_2_O, 0.24 g of KH_2_PO_4_, pH 7.4 per liter) at 4 °C. Twenty-five microliters of mPEG-maleimide reaction buffer (PBS containing 4 mM mPEG-maleimide) was added to 25 μl of 10 μM protein solutions. After incubation at 37 °C for 30 min, 5 μl of 2-mercaptoethanol was added to quench the reaction, and then 5 μl of 10% SDS was added. After centrifugation (20,000*g*, 20 min, 4 °C) to remove any aggregates, 60 μl of each soluble solution was mixed with 60 μl of 2× SDS loading buffer. After boiling at 95 °C for 3 min, each protein solution was run on SDS-PAGE and then analyzed by CBB staining.

### X-ray crystallographic study of FlhA_C_(E351A/D356A)

Initial crystallization screening was performed at 20 °C by the sitting-drop vapor-diffusion method using Wizard Classic I and II, Wizard Cryo I and II (Rigaku Reagents, Inc.), Crystal Screen, and Crystal Screen 2 (Hampton Research). Crystals suitable for X-ray analysis were obtained from drops prepared by mixing 0.5 μl protein solution with 0.5 μl reservoir solution containing 0.1 M Tris-HCl, pH 8.5, 20% (v/v) PEG 8000, and 200 mM MgCl_2_. X-ray diffraction data were collected at synchrotron beamline BL41XU in SPring-8 (Harima, Japan) with the approval of the Japan Synchrotron Radiation Research Institute (JASRI) (Proposal No. 2016B2544 and 2018A2568). The FlhA_C_(E351A/D356A) crystal was soaked in a solution containing 90% (v/v) of the reservoir solution and 10% (v/v) glycerol for a few seconds and was directly transferred into liquid nitrogen for freezing. The X-ray diffraction data were collected at the wavelength of 1.000 Å under nitrogen gas flow at 100 K. The diffraction data were processed with MOSFLM^43^ and were scaled with Aimless^44^. The initial phase was determined by molecular replacement using the software package Phenix^45^ with the wild-type FlhA_C_ structure in the orthorhombic crystal form (PDB code: 6AI0) as a search model. The atomic model was constructed with Coot^46^ and refined with Phenix^45^. During the refinement process, iterative manual modification was performed. The Ramachandran statistics indicated that 96.0%, 3.9%, and 0.1% residues were in the most favorable, allowed, and outlier regions, respectively. The diffraction data statistics and refinement statistics are summarized in Table 1.

### Statistics and reproducibility

Statistical tests, sample size, and number of biological replicates are reported in the figure legends. Statistical analyses were done using KaleidaGraph software (HULINKS). Comparisons between datasets were performed using a two-tailed Student’s *t*-test. A *P* value of <0.05 was considered to be statistically significant difference. *P < 0.05; **P < 0.01; ***P < 0.001.

### Reporting summary

Further information on research design is available in the Nature Research Reporting Summary linked to this article.

## Supplementary information

Peer Review File

Supplementary Information

Description of Additional Supplementary Files

Supplementary Data 1

Reporting Summary

## Data Availability

The X-ray crystal structure and structure factors of FlhA_C_(E351A/D356A) have been deposited in Protein Data Bank under the accession code 7CTN. All data generated during this study are included in this published article, Supplementary Information and Supplementary Data file. Strains, plasmids, polyclonal antibodies, and all other data are available from the corresponding author on reasonable request.
