# Supplementary material for: The FlhA linker mediates flagellar protein export switching during flagellar assembly
Source: Commun Biol. 2021 May 31;4:646. doi: 10.1038/s42003-021-02177-z (PMC8166844; doi:10.1038/s42003-021-02177-z)
Supplement: Supplementary file 2 — Supplementary Information [file 42003_2021_2177_MOESM2_ESM.pdf]

Supporting Information

The FlhA linker mediates flagellar protein export switching during flagellar assembly

Yumi Inoue, Miki Kinoshita, Mamoru Kida, Norihiro Takekawa, Keiichi Namba, Katsumi Imada and Tohru Minamino

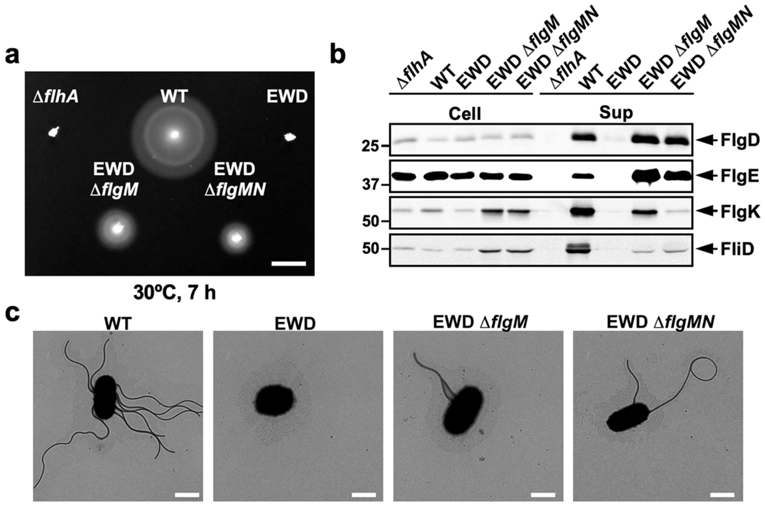

Supplementary Fig. 1. Isolation of pseudorevertants from the *flhA*$_{EWD}$ mutant. (a) Motility of the *Salmonella* NH001 strain transformed with pTrc99AFF4 (Δ*flhA*), pMM130 (WT), or pYI003 [FlhA(E351A/W354A/D356A) indicated as EWD]), YI1003-4 (EWD Δ*flgM*) or YI1003-13 (EWD Δ*flgMN*) in soft agar. Scale bar, 1.0 cm. **(b)** Immunoblotting using polyclonal anti-FlgD (1st row), anti-FlgE (2nd row), anti-FlgK (3rd row) or anti-FliD (4th row) antibody, of whole cell proteins (Cell) and culture supernatants (Sup) prepared from the above strains. **(c)** Electron micrographs of the above cells. Scale bar, 1.0 µm.

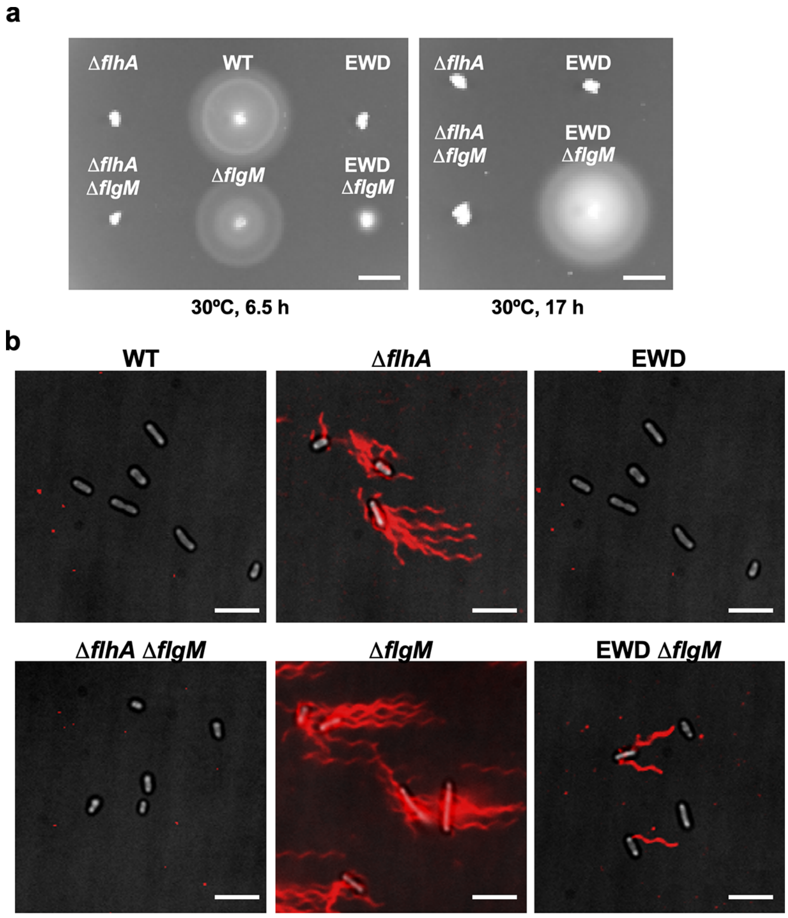

Supplementary Fig. 2. Effect of the Δ*flgM*::*km* allele on motility of the *flhA*$_{EWD}$ mutant. **(a)** Motility of the *Salmonella* NH001 strain transformed with pTrc99AFF4 (Δ*flhA*), pMM130 (WT), or pYI003 (EWD) and the *Salmonella* NH001gM strain carrying pTrc99A (Δ*flhA* Δ*flgM*), pMM130 (Δ*flgM*), or pYI003 (EDW Δ*flgM*) in soft agar. Scale bar, 1.0 cm. **(b)** Fluorescent images of the above transformants. Flagellar filaments were labelled with Alexa Fluor 594. The fluorescence images of the filaments labelled with Alexa Fluor 594 (red) were merged with the bright field images of the cell bodies. Scale bar, 5.0 µm.

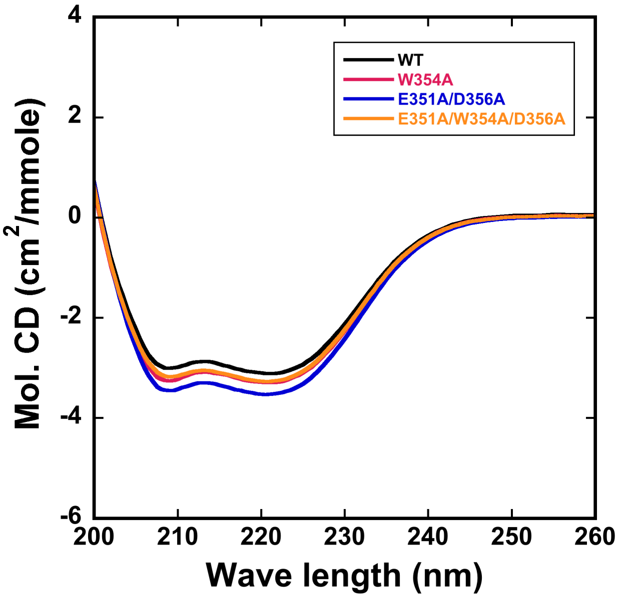

Supplementary Fig. 3. Effect of FlhA linker mutations on far-UV CD spectra of FlhA$_C$. Measurements were carried out at room temperature in 20 mM Tris-HCl, pH 8.0, in a quartz cell with a path length of 1 mm.

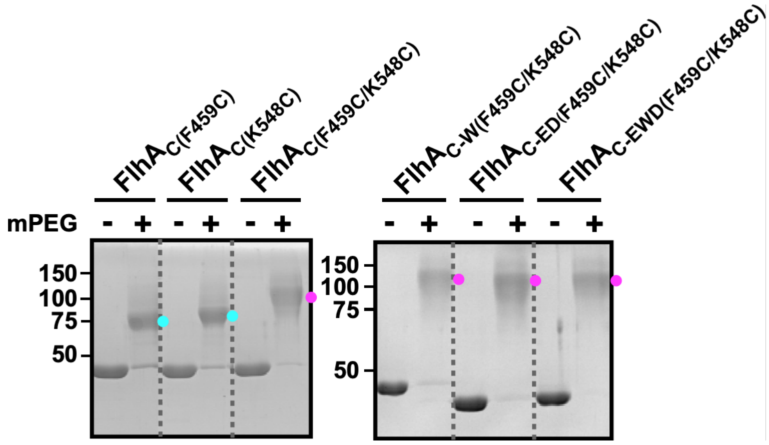

Supplementary Fig. 4. Effect of FlhA linker mutations on mPEG-maleimide modification of Cys-459 and Cys-548. His-FlhA$_{C(F459C)}$, His-FlhA$_{C(K548C)}$, His-FlhA$_{C(F459C/K548C)}$, His-FlhA$_{C-W(F459C/K548C)}$, His-FlhA$_{C-ED(F459C/K548C)}$ and His-FlhA$_{C-EWD(F459C/K548C)}$ were incubated with (+) or without (-) mPEG-maleimide. After centrifugation at 20,000 g for 20 min to remove any aggregates, supernatants were analyzed by SDS-PAGE with CBB staining. Cyan and magenta dots indicate positions of FlhA$_C$-(mPEG) and FlhA$_C$-(mPEG)$_2$, respectively.

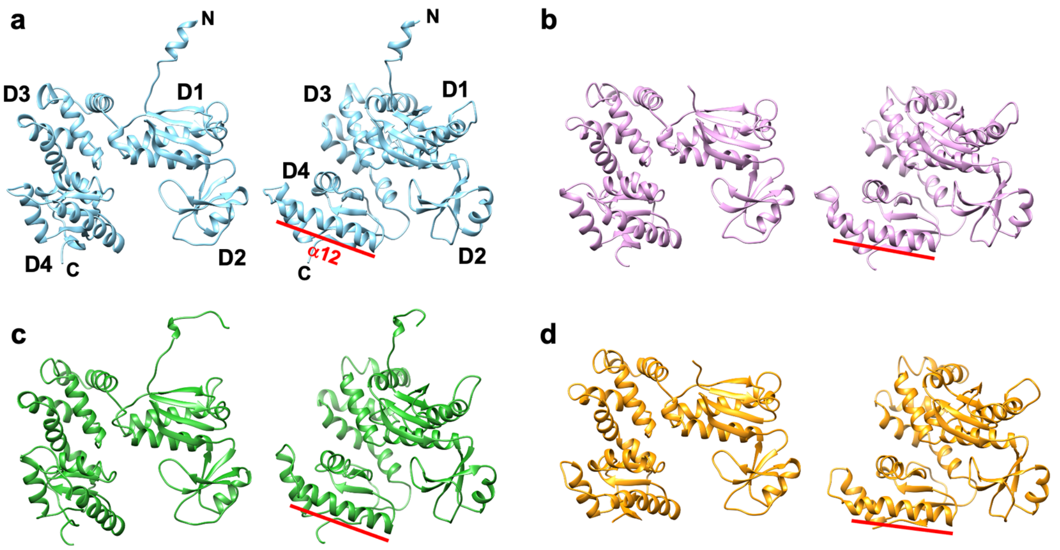

Supplementary Fig. 5. **Two distinct conformations of FlhA$_{C\text{-}ED}$ in the crystal.** Ribbon representations of the crystal structures of **(a)** Mol-A of FlhA$_{C\text{-}ED}$, **(b)** Mol-B of FlhA$_{C\text{-}ED}$, **(c)** wild-type FlhA$_C$ in an open conformation (PDB code: 3A5I), and **(d)** wild-type FlhA$_C$ in a semi-closed conformation (PDB code: 6AI0). The right panel shows the view from the right side of the left panel. Red bar shows the direction of the α12 helix, which is a good indicator of the orientation of domain D4.

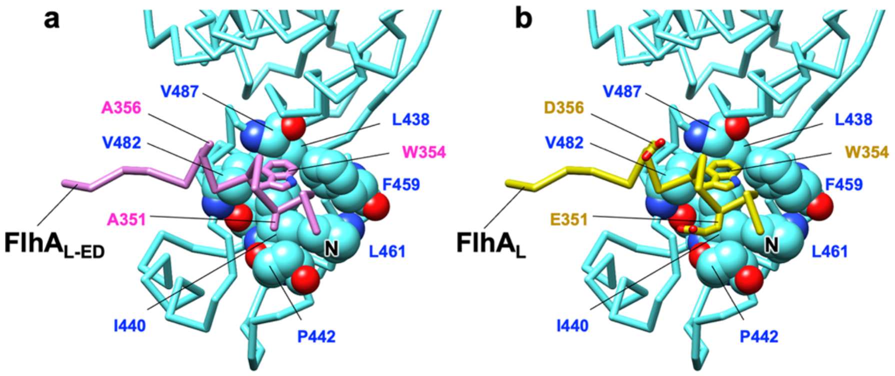

Supplementary Fig. 6. Interaction between $FlhA_L$ and a hydrophobic dimple of $FlhA_C$. **(a)** Binding of $FlhA_L$ of $FlhA_{C\text{-}ED}$ (magenta) to a well conserved hydrophobic dimple of neighboring $FlhA_{C\text{-}ED}$ (cyan) in the crystal. **(b)** Possible interaction between $FlhA_L$ of wild type $FlhA_C$ and the hydrophobic dimple. We replaced Ala-351 and Ala-356 of $FlhA_{C\text{-}ED}$ by original Glu-351 and Asp-356 residues, respectively. The side chain arm of Glu-351 is in very close to Pro-442 and so it can make a hydrophobic contact with Pro-442 to stabilize the interaction between $FlhA_L$ and the hydrophobic dimple.

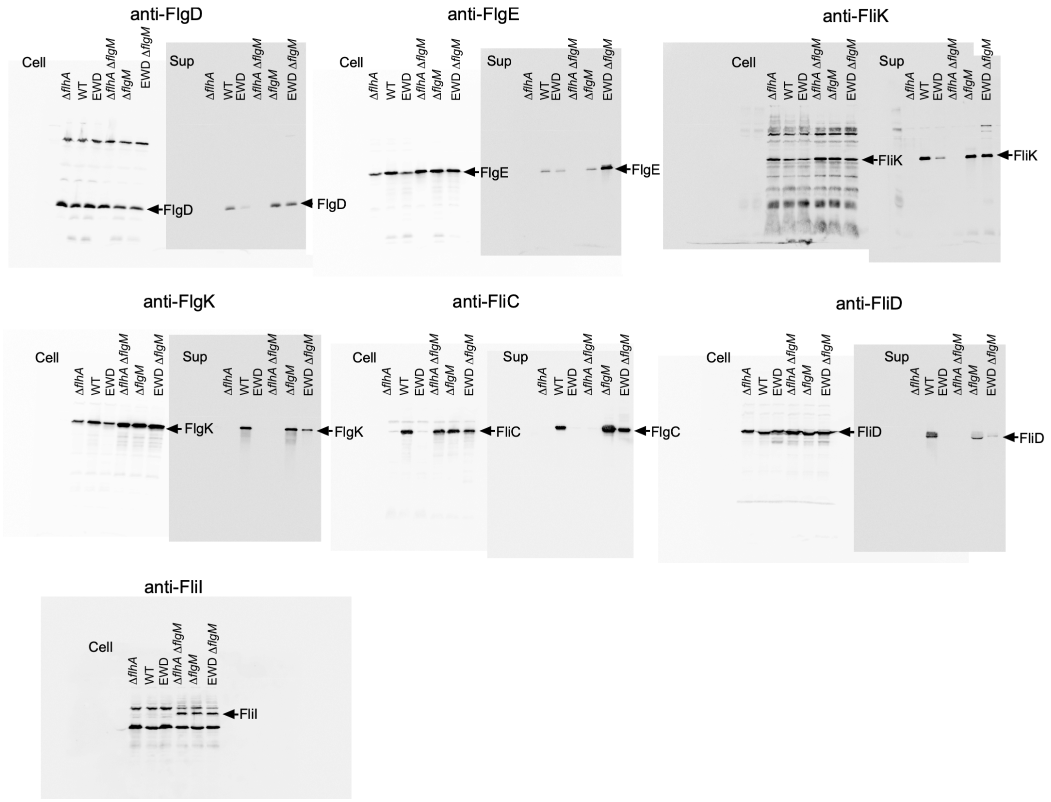

Supplementary Fig. 7. Original immunoblots shown in Figure 2b.

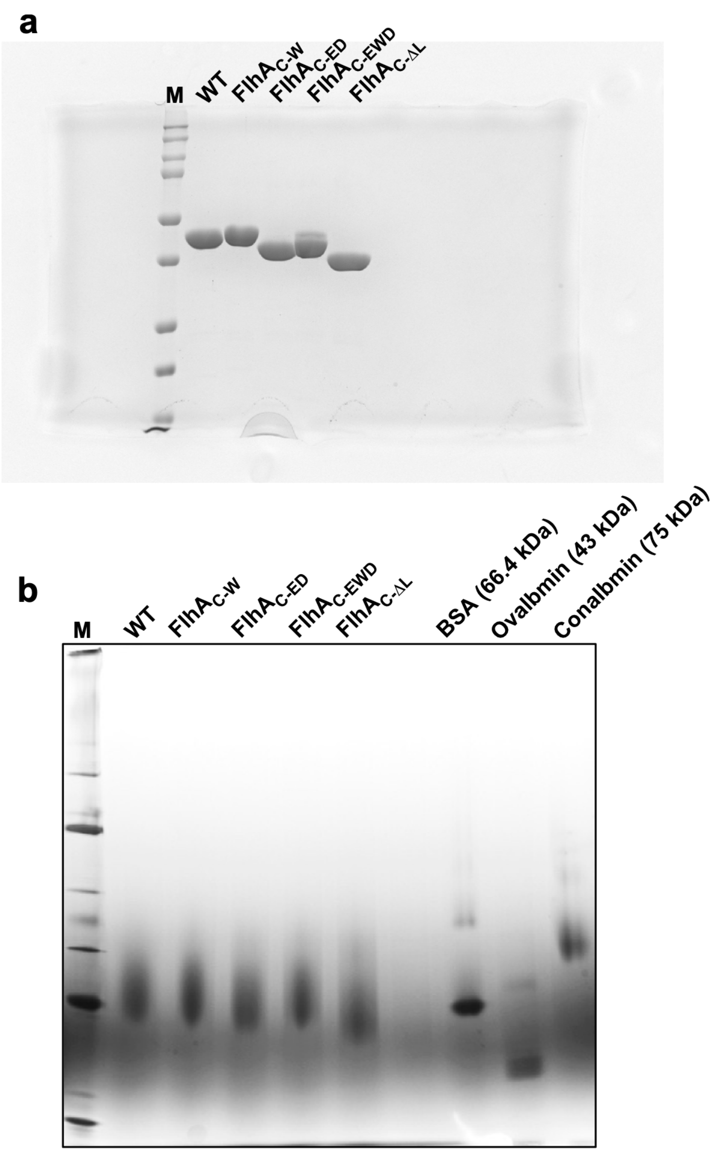

Supplementary Fig. 8. (a) Original CBB stained SDS-PAGE gel shown in Figure 5b and (b) original Blue Native PAGE gel shown in Figure 5c.
