# Supplementary material for: The FlhA linker mediates flagellar protein export switching during flagellar assembly
Source: Commun Biol. 2021 May 31;4:646. doi: 10.1038/s42003-021-02177-z (PMC8166844; doi:10.1038/s42003-021-02177-z)
Supplement: Supplementary file 3 — Description of Additional Supplementary Files [file 42003_2021_2177_MOESM3_ESM.pdf]

Description of Additional Supplementary Files

File name: Supplementary Data

Description: Source data for all graphs and charts.
